# The Application Progress of Patient-Derived Tumor Xenograft Models After Cholangiocarcinoma Surgeries

**DOI:** 10.3389/fonc.2021.628636

**Published:** 2021-07-22

**Authors:** Jun Wu, Jiyao Sheng, Hanjiao Qin, Mengying Cui, Yongsheng Yang, Xuewen Zhang

**Affiliations:** ^1^ Department of Hepatopancreatobiliary Surgery, The Second Hospital of Jilin University, Changchun, China; ^2^ Department of Radiotherapy, The Second Hospital of Jilin University, Changchun, China

**Keywords:** patient-derived tumor xenograft models, cholangiocarcinoma, target treatment, individualized treatment, chemotherapy

## Abstract

Surgical treatment is the only possible cure for cholangiocarcinoma (CCA) at present. However, the high recurrence rate of postoperative CCA leads to a very poor prognosis for patients, effective postoperative chemotherapy is hence the key to preventing the recurrence of CCA. The sensitivity of CCA to cytotoxic chemotherapy drugs and targeted drugs varies from person to person, and therefore, the screening of sensitive drugs has become an important topic after CCA surgeries. Patient-Derived tumor Xenograft models (PDX) can stably retain the genetic and pathological characteristics of primary tumors, and better simulate the tumor microenvironment of CCA. The model is also of great significance in screening therapeutic targeted drugs after CCA, analyzing predictive biomarkers, and improving signal pathways in prognosis and basic research. This paper will review the current established methods and applications of the patient-derived tumor xenograft model of cholangiocarcinoma, aiming to provide new ideas for basic research and individualized treatment of cholangiocarcinoma after surgery.

## Introduction

Cholangiocarcinoma (CCA) is divided into intrahepatic cholangiocarcinoma (ICC), hilar cholangiocarcinoma (PCC), and Distal cholangiocarcinoma (DCC) based on the anatomical location (1). CCA accounts for about 15-20% of primary hepatobiliary malignancies (2). Its early clinical manifestations are not obvious and develop rapidly, characterized by a high mortality rate, strong invasiveness and poor prognosis. Its 5-year survival rate is only 10-20% (3–5). Usually, the occurrence of CCA is related to related risk factors such as cholestatic liver disease, cirrhosis and gallstones, chronic infection (hepatitis B, hepatitis C), inflammatory disease (inflammatory bowel disease), etc. (6). At present, surgical operation is the only possible treatment for CCA, but the recurrence rate is as high as 40%-80% (3–5). Therefore, postoperative adjuvant treatment has become an effective method to improve the CCA’s recurrence.

Current studies have shown that most CCA has multiple drug resistance (MDR), and there are complex drug resistance mechanisms (7). The mechanism of CCA chemoresistance is mostly related to the following aspects: ① The decrease in drug absorption and the increase of drug efflux leading to lower intracellular drug concentration (8); ② The reduced ability of tumor cells to activate prodrugs or the increased ability to detoxify (9); ③ Molecular target mutations (10, 11); ④ DNA repair mechanism: a part of chemotherapeutic drugs exert their function by damaging DNA, but the majority of cancer cells has impaired DNA damage response, such as base excision repair system, nucleotide excision repair system or mismatch repair system (8, 12); ⑤ Regulating the death-related signaling pathways: downregulating apoptosis pathways or necrosis pathways induced by chemotherapeutic drugs (13); ⑥ Tumor microenvironment changes (14), etc. Also, research on targeted therapy in CCA is still in its infancy, with fewer useful molecular targets, and the choice of therapeutic drugs.

The rapid development of Next Generation Sequencing(NGS) has had far reaching effects in the field of CCA research. Different types of gene mutations have been found in CCA, which affect cell cycle, cytokine signal transmission, genome stability and DNA repair, including CCA-related gene mutations, amplifications, deletions, fusions and methylation (15). In ICC, genetic changes in FGFR (15.8%), IDH1 (14.7%), IDH2 (5.3%), BAP1 (14.5%), PBRM1 (12.1%), MCL1 (21.4%), CDKN2A (21.1%) are more common. And in extrahepatic cholangiocarcinoma(ECC),genetic changes in TP53 (36.9%), KRAS (31.5%), CDKN2B (15.2%), Smad4 (14.3%) are more common (16, 17). NGS undoubtedly provides a unique opportunity to expand the current understanding of CCA’s molecular pathogenesis, and it can provide individualized therapeutic targets for clinical treatment. However, there are often many differentially expressed molecules screened by sequencing technology. Therefore, using effective animal models to verify the effectiveness of screening targets has become an obstacle that limits sequencing technology to individualized treatment.

Current research in CCA has explored mechanisms of tumor development, progress, and treatment. At present, there are many CCA animal models, including CCA animal models based on tumor carcinogens (18), mouse models of bile duct ligation that mimic the characteristics of cholestasis (19), and a genetically engineered mouse model of CCA (20). But none of them can accurately predict the efficacy of conventional drugs and new anti-tumor drugs. Patient-Derived tumor Xenograft (PDX) models bring hope that better treatment options will be developed for CCA patients.

In 1985, Hudd et al. (21) used patient-derived CCA cell lines to inject subcutaneously into the abdomen of mice and successfully established a CCA CDX model. Among them, 26 of 30 mice developed CCA tumors. The CDX model is easy to develop and operate. However, due to the long-term *in vitro* culture of tumor cell lines, tumor cell lines have specific mutations, gene silencing, and lack of interaction between the tumor microenvironment and the immune system. Biological behavior and tumor heterogeneity are quite different from the original tumor tissue, which leads to an unsatisfactory prediction of clinical efficacy (22–24). Therefore, in 1984, Braakhuis and others directly implanted the head and neck tumor tissue into athymic nude mice to establish a PDX model, which stably retained the primary tumor’s genetic characteristics and histopathological characteristics (20, 25, 26). Cavalloni et al. (27) used patient-derived CCA tissue to implant subcutaneously in mice directly and successfully established a CCA PDX model for the first time, then obtained the tumor tissue by surgery, implanted the second group of mice subcutaneously to form F2 generation, and so on. They observed that the F4 generation CCA PDX showed the same morphology, histology and immunohistochemistry as the primary tumor.The F4 CCA PDX and primary tumors had highly similar genetic and molecular profiles in gene expression, tissue arrangement, RNA expression and mutation.

The CCA PDX model based on the technology mentioned above has also been continuously used for CCA targeted drug screening, analysis of predictive markers, and CCA efficacy improvement. The PDX model of cholangiocarcinoma is of great significance and has good research value and application prospects.

## The Establishment of the CCA PDX Model

Presently, the cholangiocarcinoma (CCA) PDX model establishment method is divided into the subcutaneous transplantation model and orthotopic transplantation model. The methods of establishing subcutaneous transplantation models (28) and the orthotopic transplantation models (29) have been described in detail in the literature. Usually, the F1-F3 generations are used for reproduction, and the F3 generations are used for drug testing. The F5–F8 generations can better retain the biological and genetic characteristics of primary tumor cells. Therefore, it is advantageous to conduct drug trials in F3–F8 to predict clinical efficacy (30, 31).

The advantage of subcutaneous transplantation is the high tumor formation rate. The PDX model constructed by Vaeteewoottacharn shows a rate as high as 75% (32), in which it is easy to generate models and monitor tumor size (33). However, the disadvantage is that it cannot provide a vital microenvironment for tumor development (34).

Moreover, the common construction method of the CCA PDX model is orthotopic transplantation. By implanting human intrahepatic CCA (ICC) tumor tissue into the liver of mice, an orthotopic CCA transplantation model is generated. Orthotopic transplantation can provide a better original tumor microenvironment than conventional subcutaneous transplantation, but it is relatively rare due to its difficulty and low tumor formation rate (20). Orthotopic transplantation of hilar and distal CCA is rare due to the difficulty of operation. There are several factors that affect the success of the model, such as transplantation within 60 min. If transplantation cannot be performed immediately or the first transplantation fails within 60 min, the loss can be reduced by cryopreservation technology. For the first time, Hernandez et al. established a PDX model of CCA within 60 min. Seventeen cases of CCA PDX models showed failed implantation. Then, 17 cases of CCA were immediately cryopreserved and implanted with resuscitation. In mice, 12 cases (70%) of PDX models of CCA were successfully constructed under the skin, which proved that long-term preservation of CCA could be achieved using cryopreservation technology (35).

Conversely, the success of PDX establishment varies with tumor origin and disease characteristics. More aggressive, recurrent, and highly metastatic tumors tend to show higher transplantation rates (36). According to reports, the KRAS mutation may be the key gene for its success (27).

## Application of CCA PDX Model in Targeted Therapy

**Figure 1:**
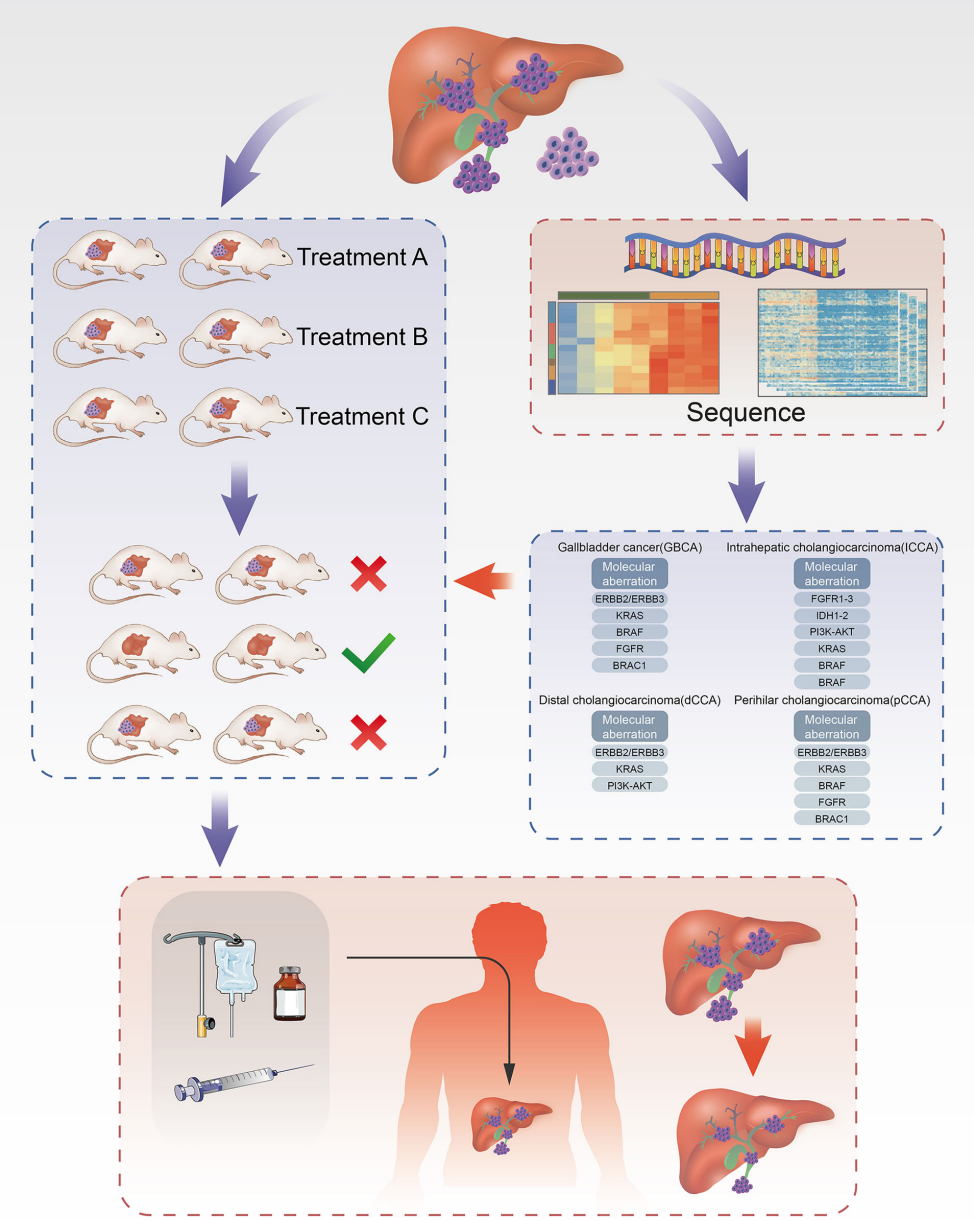
Establishment of ICC PDX models and application of drug screening. Part of the tissue of ICC after cholangiocarcinoma surgeries was transplanted into each group of mice correspondingly to cultivate cholangiocarcinoma. In the meanwhile, part of tissue was used for the analysis of targets for ICC using NGS technique to filter drugs with higher sensitivity and then administered on the treatment group. Through the analysis of drug efficacy against intrahepatic cholangiocarcinoma in the treatment group, the drugs with high sensitivity can be obtained and further acted on the human body.

In patients with advanced CCA, chemotherapy or targeted therapy is an important method to prolong overall survival. Presently, most guidelines recommend palliative treatment for patients with advanced CCA, such as chemotherapy regimens based on fluorouracil, gemcitabine, or platinum-based cytotoxic chemotherapeutics (37), but its effect is limited (38). Moreover, ICC is extremely resistant to chemotherapy drugs. Therefore, there is an urgent need for new systemic treatments to improve the prognosis of ICC. To date, a large number of studies have been conducted on CCA, and the mechanism of its development has been explored and understood, while various related targets have been discovered, for example, proto-oncogene c-Myc, fibroblast growth factor receptor, KRAS gene, Hippo-YAP signaling pathway, ubiquitin proteasome pathway, Notch signaling pathway, cyclin-dependent kinase family, and vascular endothelial growth factor (24, 39). However, none of the studies have shown targeted therapy that has advantages over systemic cytotoxic chemotherapy (38), and the reason is that there is no individualized screening of targeted drugs for CCA. Thus, exploration of CCA targets is still needed. The emergence of the PDX model links basic medical research to clinical trials. In contrast, the PDX model can be used for extensive sensitivity screening before targeted drugs. Moreover, the PDX model can be used to further explore the mechanism and development of new therapeutic targets (**Figure 1**).

### Application of CCA PDX Model in Targeted Drug Screening

#### ADRM1 Inhibitor

The PDX model has been used to a certain extent in the screening of targeted drugs for cholangiocarcinoma (**Table 1**). The 26S proteasome is the main component of the ubiquitin-proteasome pathway responsible for regulating the protein degradation of more than 80% of mammalian cells (62). The ubiquitin-proteasome pathway degrades some apoptosis-related proteins through the 26S proteasome and regulates cell apoptosis. Studies have shown that actively proliferating malignant tumor cells are more sensitive to the ubiquitin-proteasome pathway inhibitors than normal cells (63). Using inhibitors against the ubiquitin-proteasome pathway is a new and powerful strategy for the treatment of malignant tumors.

**Table 1:** CCA PDX models.

Mouse strain	Tumor source	Planting pattern	Incision site	Implanted tumor volume	Target screen	Drug screen	References
NOD/Shi-SCID female mice	ICC	Subcutaneous transplantation	–	–	–	Ecteinascidin-743	(40)
NOD/SCID/Il2rg null (NSG) mice	Metastatic lung nodule from IV ICC	Subcutaneous transplantation	Right flank	–	FGFR	BGJ398	(41)
NOD/SCID mice	CCA	Subcutaneous transplantation	In the flank area in the middle of the thigh line	–	FGFR	BGJ398	(42)
NOD/Shi-SCID mice	ICC	Subcutaneous transplantation	Subcutaneous	4x4 mm	KRAS	–	(27)
NOD/SCID mice	ICC	Subcutaneous transplantation	–	–	SYK, LGALS1	Trabectedin	(43)
CB17-/- SCID mice	ECC	Subcutaneous transplantation	Right flank	5x5x5 mm	c-Myc	JQ1	(24)
CB17SC-M-F scid−/− mice	ICC	Subcutaneous transplantation	The base of the tail	–	ADRM1	RA190	(44)
NOD/SCID mice	CCA	Subcutaneous transplantation	In the flank area in the middle of the thigh line	–	Hippo-YAP	Dasatinib	(45)
NOD/SCID mice	ICC	Subcutaneous transplantation	In the flank area in the middle of the thigh line	1x1x1mm	FGFR	LY2874455	(46)
NOD/SCID mice	CCA	Subcutaneous transplantation	In the flank area in the middle of the thigh line	–	SFK	SFK inhibitor	(47)
Balb/c Rag-2 -/-Jak3 -/- mice	CCA	Subcutaneous transplantation	–	–	CDK4/6-pRB	CDK4/6 inhibitor	(48)
NOD/SCID mice	CCA (stage IV)	Subcutaneous transplantation	Flanks	3x3 mm	Bcl-xl-miR-876	–	(49)
NOD/SCID mice	CCA	Subcutaneous transplantation	Bilateral subcutaneous pockets	1–2 mm fragments	–	–	(50)
Balb/c nude Rag-2/Jak3-deficient (Nude RJ) mice	ICC, ECC	Subcutaneous transplantation	Flanks	2-3 mm	–	–	(32)
BALB/c nude mice	CCA	Orthotopic transplantation	The liver at a depth of 3 mm	–	–	–	(29)
NOD/SCID/Il2rg-knockout (NSG) mice	ICC	Subcutaneous transplantation	Right flank	–	FGFR2-CCDC6	Ponatinib	(51)
NOD/SCID mice	CCA	Subcutaneous transplantation	Flanks	3x3x3 mm	VEGFR2	Anlotinib	(52)
NOD/SCID mice	CCA	Subcutaneous transplantation	Subcutaneous	3 mm^3^	PTEN-proteasome	Bortezomib	(53)
NOD/SCID mice	CCA	Subcutaneous transplantation	Bilateral flanks	1x1x1mm	–	–	(54)
NOD/SCID mice	CCA	Subcutaneous transplantation	Bilateral flanks	1–2mm fragments	–	NUC1031	(55)
NOD scid gamma (NSG) mice	CCA	Subcutaneous transplantation	Flanks	3x3 mm	CDK2/5/9	Dinaciclib	(56)
CD1 immunodeficient nude female mice	ICC	Subcutaneous transplantation	Subcutaneously in the flanks	–	Notch1	LY3039478	(57)
Female BALB/c (nu/nu) nude mice	ICC	Subcutaneous transplantation	Right flank	–	CDK7	THZ1	(58)
Male athymic nude mice	CCA	Subcutaneous transplantation	Flanks	1–2 mm^3^	PLVAP	Anti-PLVAP antibody	(59)
NOD/SCID mice	ICC	Subcutaneous transplantation	Subcutaneously in the flanks	4x4 mm	–	–	(60)
Male NOD-Prkdcscid IL2rgtm1/Bcgen mice	ICC	Subcutaneous transplantation	Subcutaneous space of the upper left flank	–	TAN, TAM	–	(61)

c-Myc,cellular-myelocytomatosis viral oncogene; CDK7, cyclin-dependent kinase 7; FGFR, Fibroblast Growth Factor Receptor; bcl-xl, B-cell lymphoma-xl; ADRM1, adhesion regulating molecule 1; CCDC6, coiled-coil domain containing 6; VEGFR2, Vascular Endothelial Growth Factor Receptor 2; PTEN, Phosphatase and tensin homologue deleted on chromosome 10; PLVAP, Recombinant Plasmalemma Vesicle Associated Protein; TAN, Tumor-associated neutrophil; TAM, Tumor-associated macrophage.

The latest research shows that the ubiquitin receptor ADRM1 is overexpressed in liver cancer, gastric cancer, ovarian cancer and other malignant tumors. And gene knockout ADRM1 can inhibit the growth of malignant tumors. RA190 is a new small molecule ADRM1 inhibitor, confirmed to have a significant inhibitory effect on multiple myeloma (64–66). Yu et al. (44) have found through *in vitro* experiments that RA190 significantly inhibits the proliferation of primary ICC cells and ICC cell lines. To further explore the application value of ADRM1 inhibitor RA190 in ICC therapy, ICC PDX models were established by subcutaneous transplantation in mice.

Yu et al. (44) established the ICC PDX model and found that RA190 could significantly reduce the volume of ICC *in vivo* suggesting ADRM1 to be a promising anti-tumor target in ICC. In the study, ICC PDX mouse models were randomly divided into two groups (n=8), and they were given RA190 and buffer, respectively. After four weeks, the results showed that the tumor size and weight in the RA190 treatment group were significantly smaller than the buffer group. Also, the RA190 treatment group showed good tolerance. RA190 showed a significant therapeutic effect on ICC by inducing G2-M cell cycle arrest and apoptosis *in vivo* and *in vitro*.

#### Notch Signaling Inhibitor

Previous studies have shown that the Notch signaling pathway plays a central role in the occurrence of cholangiocarcinoma. The activation and expression of the NOTCH1/2 receptor and the typical ligand JAGGED1 lead to the overactive Notch signaling pathway and promote ICC’s occurrence and development (67).

Mancarella et al. (57) used the ICC PDX model to find that Notch signaling pathway inhibitor LY3039478 significantly inhibits Notch pathway and growth of tumor to the same extent as gemcitabine, and clarified the mechanism of LY3039478 inhibiting the growth of cholangiocarcinoma. The study used ICC tumor tissue implanted subcutaneously in the flanks of 4–5-week-old CD1 immunodeficient nude female mice to establish an ICC PDX model, divided into three groups and received buffer, LY3039478, and gemcitabine for eight weeks. Studies showed that the level of NOTCH1 in the LY3039478 treatment group was significantly reduced. The tumor size in the LY3039478 treatment group and the gemcitabine treatment group was considerably smaller than the control group (P<0.01), and the difference was little. The tumors in the LY3039478 treatment group began to resume growth 18 days after the end of treatment, and the gemcitabine group was 11 days later. Further analysis showed that LY3039478 inhibits the Notch signaling pathway in ICC while inhibiting the expression of HES1, DLL4, VEGFA, and MMP13, confirming the significance and value of Notch signaling pathway inhibitors in ICC targeted therapy. By constructing an ICC PDX model to evaluate the efficacy of LY3039478 in a highly restored human ICC tumor environment, inhibiting the Notch signaling pathway may become a new strategy for targeted treatment of ICC. In addition, the authors found that DLL4, VEGFA, and MMP13 genes are direct NOTCH targets in CCA, but the mechanism of action of LY3039478 on these target molecules still needs to be further studied.

#### CDK7 Inhibitor

CDK7 is a major member of the cyclin-dependent kinase (CDK) family, regulating the G2/M and G1/S cell cycle processes. Existing studies have shown that inhibiting CDK7 activity can inhibit transcription and cell cycle progression, thereby inhibiting tumor growth. CDK7 may be a new target for ICC targeted therapy (68). THZ1 is a highly specific CDK7 inhibitor that has shown effective anti-tumor activity in small cell lung cancer, ovarian cancer and triple-negative breast cancer. Chen et al. (58) studied the role of THZ1 in ICC. After subcutaneous implantation in BALB/c (nu/nu) mice for three generations, they established CDK7 overexpressing ICC PDX. Two groups (n=6) were randomly divided, and they were given intraperitoneal injections of PBS and THZ1 for 17 consecutive days. The results showed that the THZ1 treatment group’s tumor volume was significantly smaller than that of the PBS group, and THZ1 did not affect the weight and liver and kidney function of the mice. This study confirmed that CDK7 might be a feasible target for ICC targeted therapy.

### Application of CCA PDX Model in the Targeted Therapy Mechanism

#### The Role of Fibroblast Growth Factor Receptor in CCA

Fibroblast growth factor receptor (FGFR) regulates cell proliferation and invasion, angiogenesis and tissue development in the body, and the fusion, rearrangement, translocation and amplification of the FGFR gene are closely related to the occurrence and development of tumors (69). In cholangiocarcinoma, FGFR2 mutations are as high as 20%, and up to 13% of ICCs have fibroblast growth factor receptor 2 (FGFR2) fusion genes (70).

Wang et al. (41) selected a fresh lung metastatic nodule tissue resected from a patient with stage IV ICC, and RNA sequencing showed that the tissue had FGFR2-CCDC6 fusion protein. They established the metastatic CCA PDX model and treated it with Ponatinib, dovitinib and BGJ398. The average tumor volume was 412.8 ± 53.82mm^3^, 269.7 ± 24.98mm^3^ and 204.2 ± 30.13mm^3^, respectively. BGJ398 had the best effect on inhibiting tumor growth. Rizvi et al. (42) also used the CCA PDX model to reach similar conclusions, confirming the therapeutic potential of BGJ398 for CCA patients with FGFR fusion protein again. Furthermore, they explored the relationship between the Hippo signaling pathway and FGFR fusion protein from the PDX model and proposed that the transcriptional regulator YAP in the Hippo signaling pathway may be a biomarker of FGFR in CCA targeted therapy. The expression of Mcl-1 in YAP-positive CCA tumor tissues is inhibited, and YAP can up-regulate the expression of FGFR1, FGFR2, and FGFR4 in CCA. The Hippo-YAP is an important pathway that can regulate cell proliferation and apoptosis (71). Changes in this pathway are related to the pathogenesis of CCA and other malignant tumors (72). YAP is its downstream effector molecule, which can be directly phosphorylated by LATS1/2, the signaling pathway’s core component. The phosphorylated YAP is eventually degraded, losing its growth-promoting and anti-apoptotic functions.

To explore the role of the Hippo-YAP signaling pathway in the mechanism of CCA occurrence and development, Sugihara et al. (45) used mouse CCA cells and CCA PDX models to conduct *in vivo* and *in vitro* experiments. The results showed that YAP is phosphorylated on tyrosine 357 (Y357). Compared with normal human bile duct cells, YAP tyrosine phosphorylation in CCA is enhanced. At the same time, the SFK inhibitor dasatinib blocks YAP tyrosine phosphorylation and induces YAP redistribution from the nucleus to the cytoplasm and down-regulates the expression of YAP target genes. siRNA targeted knockdown studies have shown that SFK member LCK plays a crucial role in mediating YAP Y357 phosphorylation and the YAP Y357 phosphorylation pathway in LCK-mediated CCA is a potential therapeutic target. Dasatinib showed tumor suppressor effects in both *in vitro* and *in vivo* experiments. The SFK inhibitor dasatinib provides theoretical support as a targeted therapy for CCA.

Kabashima et al. (46) also evaluated the efficacy of FGFR inhibitor LY2874455 on CCA through the PDX model and explored the FGFR inhibitor’s mechanism inducing CCA cell apoptosis. The results showed that FGFR inhibitors caused Mcl-1 expression inhibition in cholangiocarcinoma cell-matrix in the PDX model, leading to cell apoptosis. The research mentioned above is based on the construction of the CCA PDX model, which provides a strong basis for the study of FGFR inhibitors as individualized treatment options for CCA patients carrying the FGFR2 fusion gene, and promotes the process of individualized treatment.

#### The Role of KRAS in CCA

In 23% to 50% of intrahepatic cholangiocarcinoma and 30% to 40% of extrahepatic cholangiocarcinoma, the KRAS gene often has mutations, which accelerate tumor progression (73, 74). Cavalloni et al. (27) implanted 17 cases of ICC tumors subcutaneously into NOD (non-obese diabetic)/Shi-SCID (severe immunodeficiency) mice. The results showed KRAS G12A mutation in the PDX model. After four months, the tumor volume reached 1000 mm^3^, and the tumor was removed and transplanted into the second generation of mice until the fourth generation. Further, using immunohistochemistry and genetic analysis techniques to evaluate biliary epithelial markers, tissue structure, genetic aberrations (including KRAS mutations), transcriptome, and microRNA profiles a high degree of gene consistency between the primary tumor and the 4th generation PDX model was confirmed. Previous studies have shown that KRAS mutations play an essential role in the metastasis of colorectal cancer (75), and speculated that KRAS mutations might be the driving factor for their successful implantation. KRAS mutation in colorectal cancer is one of the biological factors of resistance mechanism in EGFR targeted therapy (76). However, the role of KRAS mutations in the resistance mechanism of EGFR targeted therapy in CCA is still controversial. Therefore, this model may be suitable for evaluating the choice of targeted therapy drugs in CCA patients with KRAS mutations against EGFR. It improves the CCA PDX system and provides a promising platform for CCA targeted therapy to achieve precision medicine. However, the success rate of the PDX model is low (only 5.8%), which may be related to the KRAS mutation. Therefore, ways to improve the success rate of this mutant CCA is still an important question.

In summary, the targeted drugs screened by the PDX models can significantly delay the progress of CCA in mice and verify the mechanism of the development of CCA *in vitro* experiments. Although CCA PDX models are still in its infancy for drug screening and new drug targets mining at present, it has shown great potential.

## CCA PDX Model Combined With Next-Generation Sequencing Technology

Next-generation sequencing technology (NGS) has been increasingly applied to the field of tumor genomics research to promote individualized treatment in clinical oncology. It has become an effective method in tumor molecular diagnostics and targeted therapy research through efficient and accurate whole genome analysis and gene mutation detection of individual tumors. NGS technology is highly sensitive and can perform whole-genome sequencing, detect new mutations, small fragment insertion or small fragment deletion, copy number changes, and detection of gene fusion or rearrangement in small fragments (77). Javle et al. (78) used NGS technology to compare gene mutations between CCA subtypes in 412 ICC patients and 57 extrahepatic cholangiocarcinoma (EHC) patients. The analysis showed that the mutation rate in ICC of TP53 was 27%, KRAS 22%, IDH 16%, and in EHC the mutation rate of TP53 was 40%, KRAS 42%, and SMAD4 21%. NGS technology can better understand the genetic basis of the occurrence and development of CCA, and help to determine feasible treatment plans to guide the individualized treatment of CCA and accelerate the process of precision medicine.

Now in CCA research, the CCA PDX model and NGS technology are closely combined to provide new theoretical support for CCA targeted therapy, evaluate drug efficacy, and improve patient prognosis more comprehensively and accurately. The proto-oncogene c-Myc can regulate cell proliferation, apoptosis and the transcription of cell cycle-related genes. During the development of CCA, c-Myc expression is up-regulated. Down-regulating or knocking out c-Myc can reduce or inhibit the invasiveness of CCA. Bromodomain and extra terminal domain (BET) inhibitor JQ1 can down-regulate the expression of c-Myc (79–82). Although JQ1 has shown its potential in preclinical models of malignant tumors such as multiple myeloma, neuroblastoma, and pancreatic cancer (83), its usefulness in CCA requires further precise preclinical trials.

To deeply explore the significance and value of JQ1 in CCA treatment, Garcia et al. (24) used primary CCA tumors from 4 patients to establish a CCA (1–4) PDX model and evaluated the efficacy of JQ1 in CCA targeted therapy for the first time. The analysis showed that the CCA (2–4) PDX model retained the mutation type similar to the primary CCA tumor. Compared with other CCA models, JQ1 inhibited the growth of CCA2 and induced DNA damage and cell apoptosis. Immunohistochemical analysis showed that JQ1 inhibited the expression of c-Myc in the CCA2 PDX model, further indicating that for patients with CCA2 tumor origin, JQ1 may be a highly sensitive targeted therapy drug. It also proves that BET inhibitors (For example, JQ1) have a good application prospect in CCA personalized targeted therapy, worthy of further exploration.

The surgically resected CCA specimens combined with gene sequencing technology were used to screen mutated target genes. However, although clinical trials can verify whether inhibiting this target can improve patients’ prognosis and efficacy, researchers cannot effectively carry out individual therapies. Therefore, the CCA PDX model was established to highly simulate human CCA tumor environment and verify the existence of mutant targets with NGS technology. Then, the pharmacodynamic analysis was performed to promote personalized drugs and predict the prognosis of CCA patients, providing a platform with high accuracy and sensitivity for improving the efficacy of CCA and promoting the individualized treatment process. It paves the way for a deeper understanding of the occurrence and development of CCA. Based on precision medicine, CCA will gradually develop towards individualized treatment.

Comprehensive whole-exome and transcriptome sequencing has defined the genetic pattern of each CCA subtype. The technology that uses gene sequencing to screen the target of CCA drug has been relatively mature for individual patients. To date, the use of the CCA PDX model for targeted drug screening has been satisfactory in animal experiments, suggesting that the combined application of these two technologies will play an important role in the individualized clinical treatment of CCA. The ongoing registered clinical studies (ChiCTR1900024033, ChiCTR1900020978, and ChiCTR-ONC-17010678) are anticipated to provide strong backing for individualized treatment of CCA. However, it is worth considering that gene sequencing technology and PDX models for drug screening require a relatively long time in patients with advanced CCA. If the treatment target cannot be screened, treatment will be seriously delayed. Therefore, it is still recommended that patients with CCA receive palliative care based on the guidelines for CCA during the time of drug screening and testing (84).

## Deficiency of CCA PDX Model

### Insufficiency of the CCA PDX Model

The CCA PDX model can reflect the genetic characteristics, histopathology, and phenotypic characteristics of patients with CCA, but the CCA PDX model cannot simulate the interaction between tumor and immune cells in the original CCA tumor microenvironment. It is significant to ensure a high success rate of a model as soon as possible and complete drug screening in the best period widely using the PDX in CCA (22, 85).

#### Low Success Rate and Long Time

It usually takes 4–8 months to establish a preclinical PDX model for patients with advanced CCA. This is such a substantial amount of time (86). Moreover, the success rate of orthotopic transplantation is low (40%) (29).

#### Tumor Matrix Replaced by Mice

When the PDX model was used for drug screening after 3–5 sub generations, the tumor matrix was almost entirely replaced by the murine matrix, interfering with drug distribution (86). During the implantation process, the human matrix components quickly disappear and are replaced by the murine microenvironment. The tumor tissue of PDX model will have genomic instability after PDX continuous transplantation, and the expression of genes related to proliferation and angiogenesis will be up-regulated, while the expression of genes that inhibit apoptosis will be down-regulated. As a result, the sensitivity to chemotherapy and targeted therapy will be changed (87).

#### Difficulty in Obtaining Specimens

The clinical manifestations of CCA are unclear, and the diagnosis is often at an advanced stage, and the best opportunity for surgery is lost. The original CCA tumor specimens that are surgically removed are small, and sufficient transplantation volume cannot be obtained, making transplantation more difficult.

#### Difficulty of Orthotopic Transplantation

Orthotopic transplantation of hilar and distal CCA is rare due to the difficulty of operation. By implanting human ICC tumor tissue into the liver of mice, an orthotopic CCA transplantation model is generated. Orthotopic transplantation can have a better original tumor microenvironment than conventional subcutaneous transplantation, but it is relatively rare due to its difficult operation and low tumor formation rate (20). At the same time, the current transplantation method is relatively simple. Although orthotopic transplantation can provide a better original tumor microenvironment than subcutaneous transplantation, it is difficult, and the tumorigenic rate is low, so current studies on orthotopic transplantation are relatively rare.

#### Lack of Immune System

The PDX model lacks a complete immune system and is not suitable for immunotherapy research.

#### Cost of PDX Model

The cost of the PDX model is relatively high, including the mice and various targeted drugs (36).

#### PDX Finder

The PDX finder has been established globally, but there are still few CCA-related studies, with only 22 cases (www.pdxfinder.org), which is not conducive for global, multicenter collaboration research (88).

### Prospect

Current studies have confirmed that CCA’s tumor microenvironment includes the expression of immune cells (T cells, macrophages, dendritic cells and NK cells) and immune checkpoints. In recent years, Immune checkpoint inhibitor (ICI) therapy has become one of the key systemic treatments for many malignant tumors, including Programmed Cell Death-1 (PD-1) and Programmed Cell Death ligand 1 (PD-L1) and Programmed Death ligand 2 (PD-L2) checkpoint ICIs (89). However, there is no standard for immunotherapy in CCA treatment, and the PDX model lacks a complete immune system and is not suitable for immunotherapy research. At present, some scholars have established humanized mice with the immune system. Hematopoietic stem cells (HSC) derived from human was injected into the bone marrow cavity or tail vein of mice after radiation treatment of immunodeficient mice, thereby destroying the Hematopoietic function of bone marrow in the mice, making it a complete human immune system. The animal model established by implanting tumor tissue into humanized mice with the immune system is called Hu-PDX (humanized patient-derived xenograft) model (90–92). Hu-PDX model can reproduce the interaction between human tumors, the human immune system and tumor microenvironment, and has a good application prospect in preclinical research and immunotherapy drug screening. It has now highlighted its essential role and value in the immunotherapy research of malignant tumors such as liver cancer, colorectal cancer, and triple-negative breast cancer. Zhao et al. (90) established a Hu-HCC-PDX model of hepatocellular carcinoma (HCC). The results showed that the expressions of PD-1 and CTLA-4 in immune cells (especially Tc cells) were uniformly replicated in all Hu- HCCS -PDX models, which further improved the screening of immunotherapy drugs for hepatocellular carcinoma and deepened the research on the mechanism of drug resistance. Hence, it is essential to note that the role of Hu-PDX model in immunotherapy cannot be ignored. However, at present, the HU-PDX model has not been established in the immunotherapy research of CCA, which may become the research focus in the future. Promoting the research progress of the CCA PDX model in immunotherapy will provide a theoretical basis for CCA clinical trials to evaluate the efficacy and safety of anti-PD-1, PD-L1 therapy and CCA immune-microenvironment therapy, and a promising preclinical research platform for CCA immunotherapy. Simultaneously, efforts should be made to develop the next generation of humanized mice with the immune system, reflecting the characteristics of the human tumor microenvironment more accurately, and promote the acceleration of preclinical research using humanized mice models.

In addition, the next research focuses on how to shorten the time to build the PDX model. Improved transplantation methods may be used, such as ultrasound-guided fine needle aspiration. Ultrasound-guided fine needle aspiration technique can also be used for tissue acquisition (29).

The ultrasound monitor is used as a reference to push CCA cells into the liver with free hands. CCA cells from patients suspended in PBS were injected directly into the liver at a depth of 3 mm. The whole process from induction of anesthesia to recovery takes <5 min. Throughout the process, the physiological controller unit is used to monitor the animal’s heart and respiratory rate through the electrode pads on the ultrasound platform. This minimally invasive technique does not require postoperative analgesics.

Due to its minimally invasive nature, this method reduces the risk of complications in orthotopic transplantation and is fast and easy to perform. The tumor volume can be measured 1–3 weeks after injection. Compared with the traditional orthotopic transplantation model, it reduces the occurrence of inflammation and shortens the animal’s healing time. All mice survived after vaccination, which improves the survival rate of animals. Moreover, the tumor uptake rate is extremely high at 73%, and subsequent growth can be monitored longitudinally and noninvasively.

**Figure 2:**
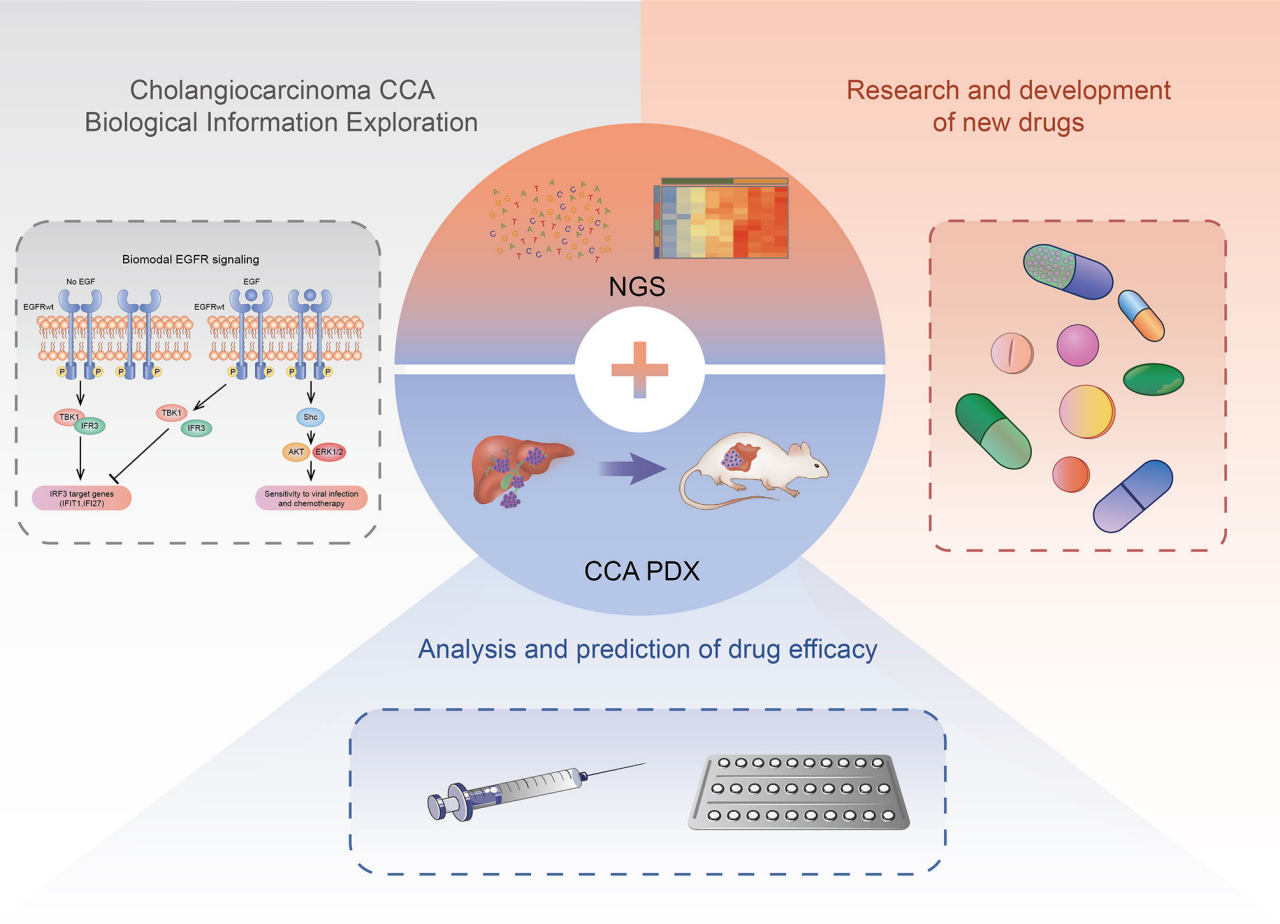
The application of CCA PDX models. The CCA PDX models have application potential in basic research, new drug development, and efficacy evaluation of the antineoplastic drug.

The current research is not entirely clear about the occurrence and development mechanism of CCA, and there is no standard for the construction of the CCA PDX model system. However, with the development of targeted therapy and immunotherapy based on precision medicine and other new treatments, the PDX model, NGS and other technologies have been applied to drug screening, efficacy evaluation, individual therapy, and basic research of CCA targeted therapy after surgery. The CCA PDX model can highly simulate the heterogeneity of human CCA tumors (**Figure 2**).

As the tumor-related animal model closest to clinical research at this stage, it has crucial translational significance for CCA preclinical research and evaluation of prognosis and good research value and application prospects. Through surgical resection and the construction of the CCA PDX model combined with targeted therapy and immunotherapy, CCA’s treatment will eventually move towards the era of individualized treatment.

## Author Contributions

JW and JS wrote the manuscript. HQ, MC, XZ and YY provided the critical revisions. All authors approved the final version of the manuscript for submission and approved it for publication.

## Funding

This work was supported by grants from China Postdoctoral Science Foundation (2020M670864), Youth Support Project of Jilin Association for Science and Technology (202028), the Project of Hepatobiliary and Pancreatic Disease Translational Medicine Platform Construction (2017F009) and Medical and Health Talents Project of Jilin Province (2019SCZT003).

## Conflict of Interest

The authors declare that the research was conducted in the absence of any commercial or financial relationships that could be construed as a potential conflict of interest.
